# Four Arguments against Noise

**Published:** 1899-02

**Authors:** 


### Four Arguments Against Noise.

The *Philadelphia Med. Journal* for July 16th says:

There are four reasons why every physician and every other good man should wage persistent war against unnecessary noises:

1. Because in a certain and increasing number of sensitive and "well" people such noises distinctly aid in carrying them over the easily passed line between comparative health and the sick and "unfit for service," thus surely increasing the sick-rate.

2. Because they decidedly destroy the vital and restorative powers of the sick, and thus clearly increase the death-rate.

3. Because they dull and brutalize the nervous systems of those who can and do learn to withstand their pathogenic influences.

4. Because they serve to make the sensitive and cultured, who are able to do so, separate themselves in their search for quiet from the masses, who must endure, thus serving to intensify the license of the noise-makers, by lessening the checks upon their crimes. The separation of the community into classes is exaggerated in this way, and these, growing wider apart, make impossible desirable helpfulness, sympathy and mutual understanding of each other. Noise is un-democratic; it should be un-American.

We endorse these sentiments and think that it might be well to begin with the suppression of the newsboy and the maddening cry of "Extry speshul! jyest aaout."

THE Department of Agriculture estimates the annual loss in 354 cities from the waste of sweepings in street-cleaning at $3,000,000.

